# Fungal inflammatory masses masquerading as colorectal cancer: a case report

**DOI:** 10.1186/s13104-014-0962-2

**Published:** 2015-02-08

**Authors:** Mohammed Iyoob Mohammed Ilyas, Sean A Jordan, Valentine Nfonsam

**Affiliations:** Department of Surgery, University of Arizona, 1501 N Campbell Avenue, Tucson, AZ 85724 USA

**Keywords:** Gastrointestinal Basidiobolomycosis, Fungal mass, Colorectal cancer

## Abstract

**Background:**

Non malignant invasive tumors of the colon and rectum are very rare. Gastrointestinal Basidiobolomycosis can present as a mass lesion mimicking colorectal cancer.

**Case presentation:**

A 56 year old Caucasian male was evaluated for abdominal and pelvic pain for 4 weeks complicated by acute urinary retention. Radiological evaluation showed him to have recto-sigmoid and cecal mass. Endoscopic examination and biopsies did not reveal a definite diagnosis. Computerized tomography guided biopsy of the mass showed fungal elements consistent with gastrointestinal basidiobolomycosis. He was treated with Itraconazole for 12 months with very good clinical and radiological response.

**Conclusion:**

Basidiobolomycosis of the gastrointestinal tract should be considered during evaluation of colorectal masses with atypical presentation. It is a rare entity seen more in endemic regions of the world for basidiobolomycosis including southwestern United States.

## Background

Rapidly progressing infiltrative lesions of colon and rectum are malignant unless proved otherwise. Benign invasive tumors of the colon and rectum are very uncommon. Gastrointestinal Basidiobolomycosis (GIB) should be considered when clinical suspicion for non malignant causes for colorectal tumors are being sought. Basidiobolomycosis is a rare but emerging cause of abdominal pain, rectal pain, and abdominal mass that is being increasingly recognized in the literature as a potential mimicker of gastrointestinal malignancy. It is caused by *Basidiobolus ranarum*, a saprophytic fungus found in the environment worldwide. Review of literature over the last two decades shows increasing reports on gastrointestinal basidiobolomycosis in immunocompetent individuals in arid regions of the world, particularly in the southwestern United States and the Middle East. We report a patient with obstructing rectal and cecal mass from invasive basidiobolomycosis which was treated without surgery. This case report and literature review would increase the awareness of GIB which is a serious but completely curable condition.

## Case presentation

**Figure 1:**
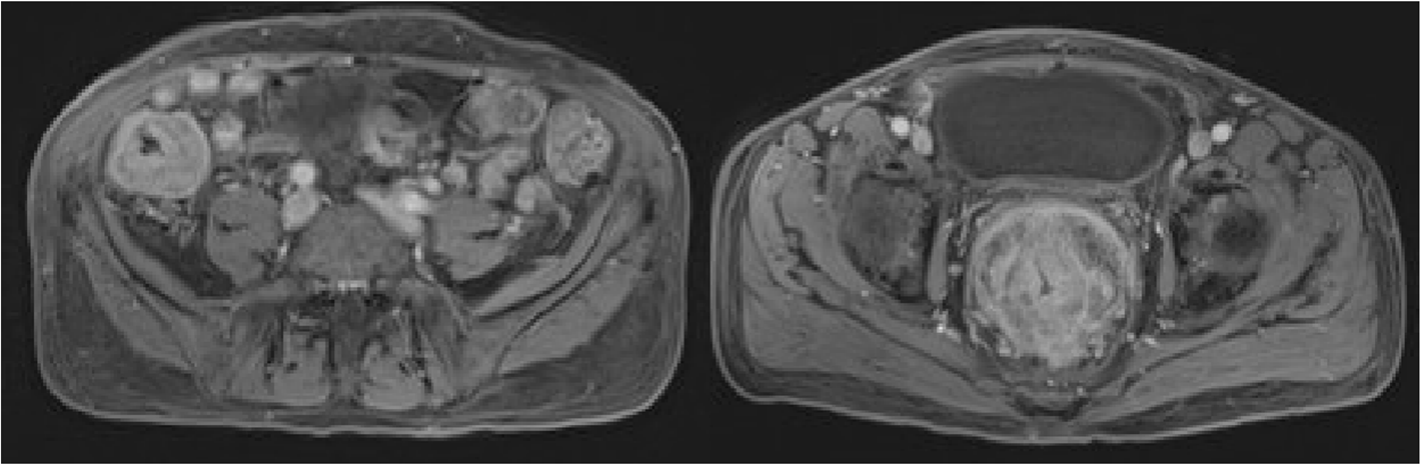
**Magnetic Resonance Imaging scan showing circumferential thickening of cecum and rectum pre treatment.**

A 56 year old Caucasian male with history of non insulin dependent diabetes presented with lower abdominal pain with increasing frequency of bowel movements for 3 months, rectal pain for a month and acute onset urinary retention. He also reported sporadic night sweats and subjective fever for 5 months. Earlier work up by his primary physician failed to elucidate a cause for his symptoms. On presentation, he was afebrile and remained hemodynamically stable. His physical examination showed right lower quadrant tenderness with no signs of peritonitis. Digital rectal examination showed tender, firm nodular mass along the anterior wall of rectum. Laboratory results revealed leukocytosis with neutrophilia and eosinophilia. Radiological examination included Computed Tomography (CT) scan and Magnetic Resonance Imaging (MRI) scan of abdomen and pelvis. They demonstrated circumferential thickening of recto sigmoid region measuring 16 cm in length with significant luminal narrowing (Figure 1). There was an extrinsic component to the lesion with extension anteriorly into seminal vesicles and prostate and posteriorly into the presacral region.

He had another inflammatory lesion involving the cecum circumferentially and measuring 6 cm in length was also noted. There were secondary inflammatory changes in the appendix and the mesentery. He had significant retroperitoneal lymphadenopathy involving the external iliac, common iliac and para-aortic nodes.

Patient’s social history was significant for his residence in southern Arizona for the last 28 years.

He is a professor of archaeology and geography with extensive travel history to the Middle East including Jordan and Israel. Incidentally he had installed a sprinkler system at his property a month prior to the onset of his symptoms. He had an earlier screening colonoscopy showing sigmoid diverticulosis 2 years ago. Differential diagnosis considered at this point included colorectal cancer and lymphoma.

**Figure 2:**
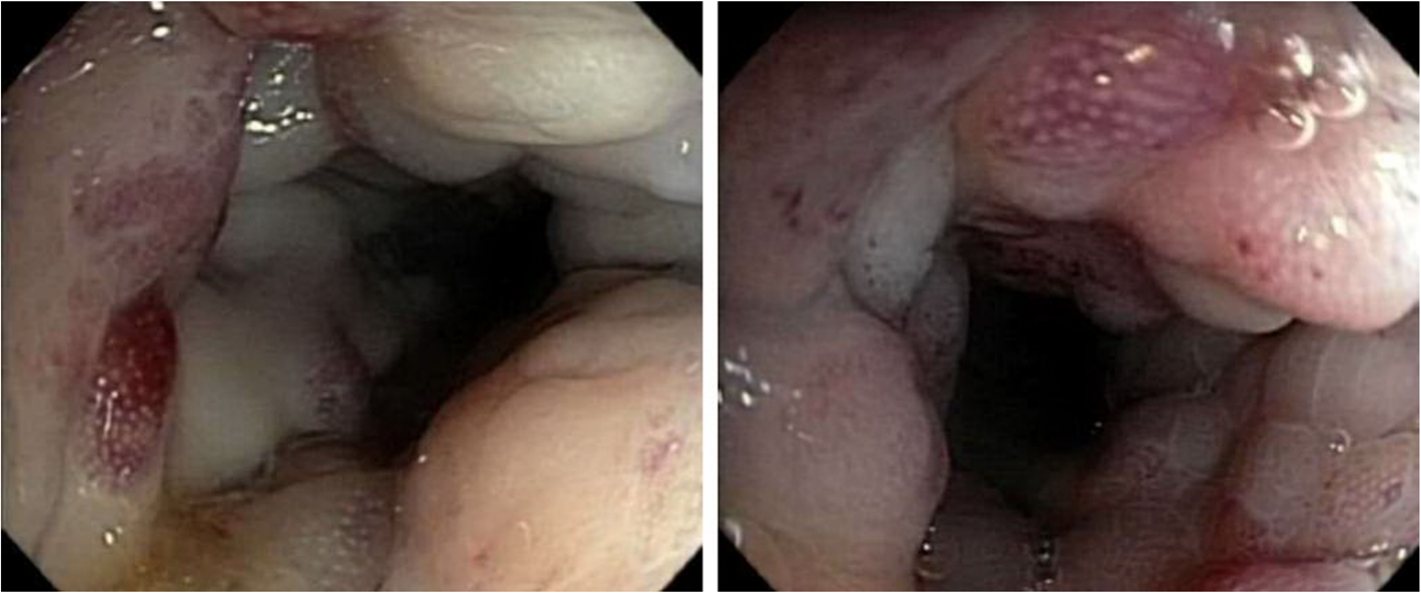
**Endoscopic examination of rectosigmoid region showing congested, friable lesion.**

**Figure 3:**
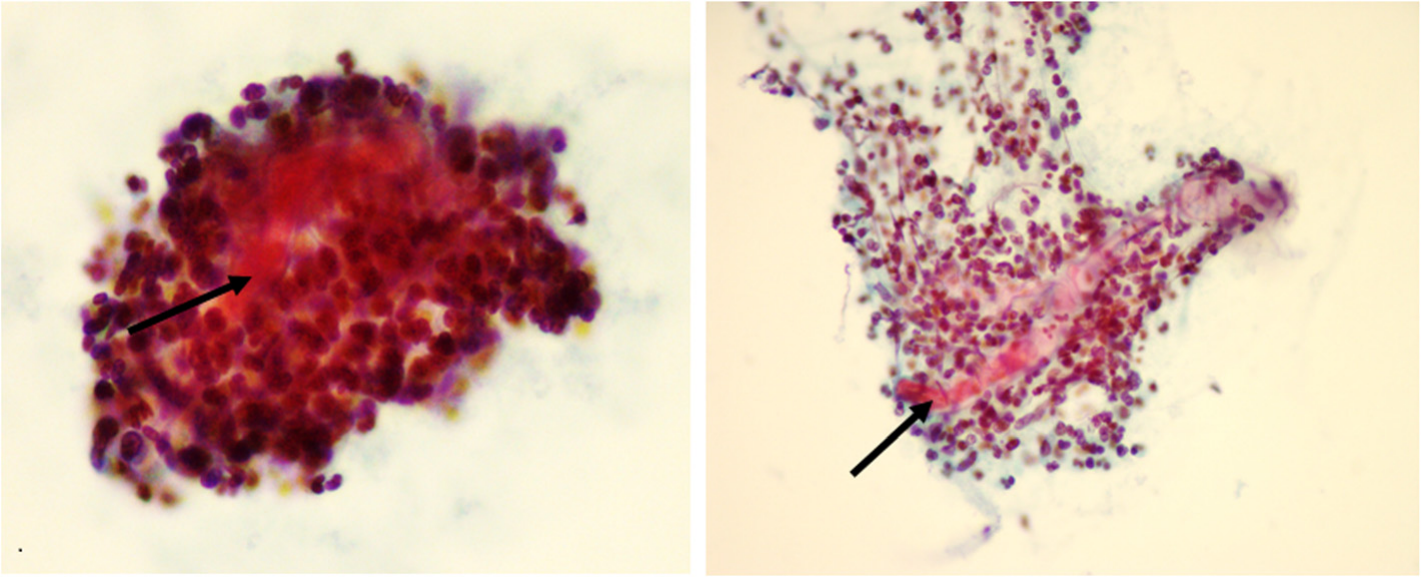
**Papanicolaou stain of rectal mass fine needle aspiration cytology with arrows showing broad non septated fungal organism surrounded by acute inflammation.**

Endoscopic examination showed congested circumferential lesion from anal verge to up to 27 cm proximal to anus (Figure 2). Histopathological examination of biopsies showed marked mucosal eosinophilia without an associated increase in mast cells and failed to reveal a specific diagnosis. Endoscopic examination and biopsies were repeated with no additional benefit. CT guided biopsy of the lesion was performed to obtain additional specimens from extrinsic component of the lesion over the cecum and recto-sigmoid lesion. Histopathological examination of CT guided biopsy showed granulation tissue with extensive acute inflammation and fibrosis along with fungal organisms which were confirmed by Grocott's methenamine silver (GMS) stain. The morphology of the fungal organisms on the staining within the clinical context were consistent with basidiobolomycosis (Figure 3). No associated malignancy was identified.

**Figure 4:**
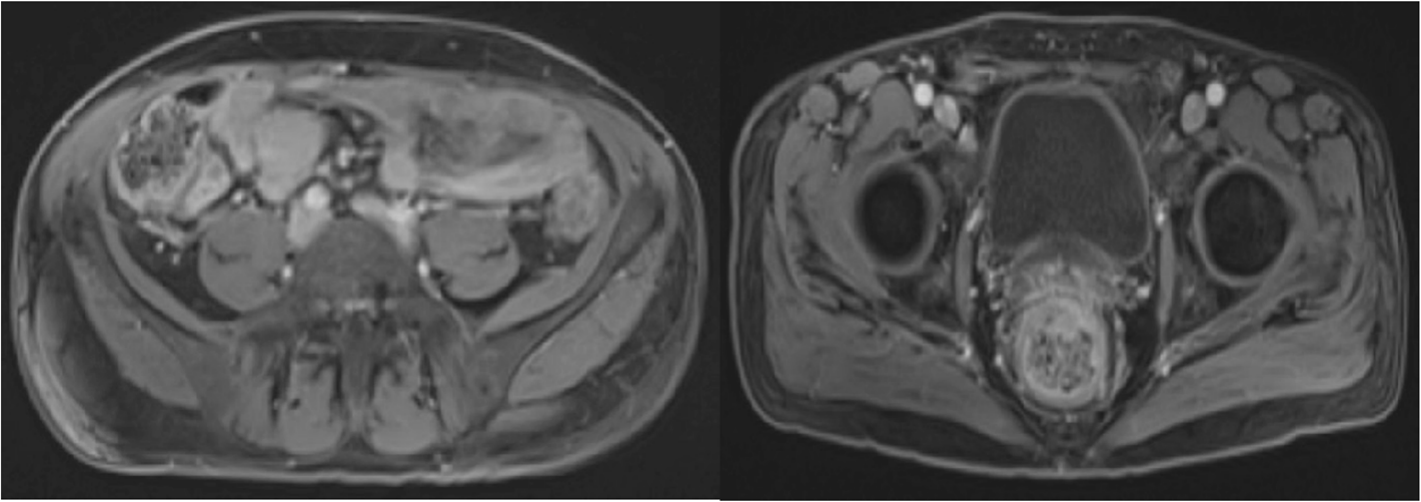
**Magnetic Resonance Imaging scan showing near resolution of the circumferential thickening of cecum and rectum after 1 year of antifungal therapy.**

The patient was treated for basidiobolomycosis with oral itraconazole therapy (100 mg oral twice daily) with significant improvement in pain, frequency of stools and urinary retention within 4 days. His anti fungal treatment was continued over the next 12 months with periodic clinical and radiological examinations. Although no specific guidelines are available on the duration of the treatment and surveillance for treatment response, we chose to evaluate him with MRI scans at 3 month intervals to evaluate the response to antifungal treatment. He continue to improve symptomatically with near resolution of the lesion on the MRI scan of pelvis after 12 months of treatment (Figure 4).

## Conclusions

Non malignant invasive tumors of the colon and rectum are very rarely seen in clinical practice. More than 95% of all invasive tumors of the colon and rectum are adenocarcinomas. Atypical presentation of lesions in the colon and rectum include squamous cell cancers, carcinoid tumors, sarcomas and lymphomas. Basidiobolomycosis of the gastrointestinal tract should be considered when both carcinomas, sarcomas and lymphoma are ruled out and other causes are being considered. It should be within the list of differential diagnosis while evaluating patients with small bowel, colonic and rectal masses of unclear origin particularly in endemic regions of basidiobolomycosis including southwestern United States.

Basidiobolomycosis is caused by *Basidiobolus ranarum*, a saprophytic fungus found in the environment worldwide. It is a member of the order Entomophthorales of the class Zygomycetes [1,2]. Clinical spectrum of diseases from Basidiobolomycosis usually include skin and subcutaneous lesions presenting as indurated lesions affecting extremities, trunk and gluteal region only [3,4]. Inoculation from minor trauma or insect bites appear to be the mode of acquisition of the fungus [5-7]. Unintentional ingestion of the fungus from contaminated soil or fruits or vegetables is touted as the route of entry preceding GIB. Although living in tropical and subtropical areas are well established as risk factors for basidiobolomycosis, no risk factors are identifiable for GI basidiobolomycosis per se. Incidentally, basidiobolomycosis affects men more likely due to their indulgence in outdoor activity compared to women in the tropics.

Literature review by Vikram *et al*. [8] showed 44 cases of GIB in the previous 30 years [8]. Most of the cases reported are from Arizona and Saudi Arabia. Most of these patients were diagnosed with abdominal mass on CT scan and endoscopy after having been evaluated for indolent non specific signs and symptoms. Patients were normally immunocompetent men with no underlying serious medical problems. Routine laboratory work up showed peripheral eosinophilia. Diagnosis were confirmed by culture of tissue specimens. GIB involved colon (82%); small bowel (36%) and liver and gallbladder (30%). 84% patients underwent surgical excision of the masses and were treated with antifungal agents. 80% of patients survived following the above management.

There have been 17 previous reports of GIB from Arizona since 1997. Two other cases have been reported from Utah and Florida [9]. Case control study conducted by Center for Disease Control and Prevention (CDC) showed the following potential risk factors for GIB: Prolonged residence in Arizona, prior treatment of peptic ulcer disease, diabetes, outdoor activities including gardening and landscaping [10].

Case series by Saud Al Shanafey reported 9 pediatric cases of GIB at King Faisal Specialist Hospital and research center between 2001 and 2010 [11]. Distribution of lesions included left colon involvement in 11%, right colonic involvement in 33%, liver involvement in 78% and diffuse abdominal disease in 22%. Patients with colonic involvement in GIB had right or left hemicolectomy. Liver lesions were managed with partial hepatectomy. One patient who had multiple smaller lesions in both lobe of the liver was treated with antifungal medications successfully. It is interesting to note that 89% of the patients were of male gender. These patients had a median workup for 4 months before being diagnosed with GIB. Clinical presentation generally included vague abdominal pain, fever, change in bowel habits, weight loss and palpable abdominal mass. Tests showed patients to have leukocytosis, eosinophilia, raised ESR and CRP. Two patients had disseminated abdominal disease and died despite aggressive management. All the surviving patients were treated with long term antifungal medications. Pediatric GIB has also been reported by Al Jarie *et al.* and others [12-14].

Management of GIB depends on the clinical presentations. Patients who present with complete bowel obstruction would need emergent surgery to relieve the obstruction. Failure to identify and appropriately treat this rare diagnosis has been complicated by acute colonic obstruction necessitating emergency colostomy [13] and mortality [15]. Sub acute to chronic presentations (as in our patient) could be managed electively. Although Saud Al Shanafey *et al.* proposed early surgical intervention in patients with GIB to avoid morbidity and mortality, it may not be necessary when the presentation is rather less emergent. Anti fungal treatment alone may be sufficient to eradicate the lesion (as in our patient), but it would depend on the clinical presentation and the response of the lesion to antifungal treatment. If surgery is required, conservative resection to reduce the bulk of the lesion and to maintain GI and biliary continuity would be sufficient. This has been reported to improve response to medical management. Close follow up is recommended in the postoperative period to ensure eradication of the disease. Investigations during the follow up period would depend on the mode of presentation and the site of the lesion. Response to medical treatment of lesions involving the cecum, ascending colon and recto-sigmoid region can be evaluated with MRI of abdomen and pelvis.

Duration of treatment has not been standardized. Most of the patients have been treated with antifungal treatment ranging from 6 months to 1 year. Vikram *et al.* reported relapse with discontinuation of treatment after 3 months based on symptomatic improvement.

Diagnosis of basidiobolomycosis require culture of B ranarum from the tissue specimen. But, presumptive diagnosis could be made from the histopathological appearance. Microscopic appearance of B ranarum include scarce, broad and thin walled pleomorphic hyphae surrounded by collar of eosinophilic material known as Splendore­hoeppli phenomenon [16]. Susceptibility of Basidiobolomycosis to antifungal agents are known to be highly variable and isolate dependent [17,18]. Empiric treatment could be started with Itraconazole or Voriconazole, but susceptibility testing is recommended to ensure effective treatment. Taghipour *et al.* reported a mortality with GIB from septic shock and pulmonary insufficiency despite successful diagnosis and treatment with Itraconazole and amphotericin B. Surveillance of treatment response could be performed with MRI or CT scans. There are no consensus opinion on the imaging modality and duration of surveillance in the literature. We propose the surveillance imaging could be performed with CT scan or MRI scans depending on the local availability and expertise.

In summary, GIB is a rare invasive fungal infection involving the gastrointestinal tract and liver. If identified early and treated appropriately, it is curable and surgery can be avoided. Surgery is unavoidable if GIB is complicated by bowel obstruction. Awareness and knowledge is important for early diagnosis and treatment of GIB and prevent morbidity and mortality from delay in diagnosis.

## Consent

Written informed consent was obtained from the patient for publication of this Case Report and any accompanying images. A copy of the written consent is available for review by the Editor-in-Chief of this journal.

## Abbreviations

GIB: Gastrointestinal Basidiobolomycosis
CT: Computerized tomography
MRI: Magnetic Resonance Imaging
GMS: Grocott’s methenamine silver stain
CDC: Center for Disease Control and Prevention

## References

[1] Hussein MR, Musalam AO, Assiry MH, Eid RA, El Motawa AM, Gamel AM (2007). Histological and ultrastructural features of gastrointestinal basidiobolomycosis. Mycol Res.

[2] Kwon­Chung KJ, Bennett JE, Lea and Febiger. Medical mycology*:* 1992;449–63.

[3] Bittencourt AL, Ayala MA, Ramos EA (1979). A new form of abdominal zygomycosis different from mucormycosis: report of two cases and review of the literature. Am J Trop Med Hyg.

[4] Khan ZU, Prakash B, Kapoor MM, Madda JP, Chandy R (1998). Basidiobolomycosis of the rectum masquerading as Crohn's disease: case report and review. Clin Infect Dis.

[5] Sujatha S, Sheeladevi C, Khyriem AB, Parija SC, Thappa DM (2003). Subcutaneous zygomycosis caused by Basidiobolus ranarum: a case report. Indian J Med Microbiol.

[6] Bittencourt AL, Marback R, Nossa LM (2006). Mucocutaneous entomophthoramycosis acquired by conjunctival inoculation of the fungus. Am J Trop Med Hyg.

[7] Kamalam A, Thambiah AS (1982). Basidiobolomycosis following injection injury. Mykosen.

[8] Vikram HR, Smilack JD, Leighton JA, Crowell MD, De Petris G (2012). Emergence of gastrointestinal basidiobolomycosis in the United States, with a review of worldwide cases. Clin Infect Dis.

[9] Schmidt JH, Howard RJ, Chen JL, Pierson KK (1986). First culture proven gastrointestinal entermophthoromycosis in the United States: a case report and review of the literature. Mycopathologia.

[10] Lyon Lyon GM, Smilack JD, Komatsu KK, Pasha TM, Leighton JA, Guarner J, Colby TV, Lindsley MD, Phelan M, Warnock DW, Hajjeh RA (2001). Gastrointestinal basidiobolomycosis in Arizona: clinical and epidemiological characteristics and review of the literature. Clin Infect Dis.

[11] Al Shanafey S, AlRobean F, Bin Hussain I (2012). Surgical management of gastrointestinal basidiobolomycosis in pediatric patients. J Pediatr Surg.

[12] Al Jarie A, Al Mohsen I, Al Jumaah S, Al Hazmi M, Al Zamil F, Al Zahrani M, Al Modovar E, Al Dayel F, Al Arishii H, Shehrani D, Martins J, Al Mehaidib A, Rossi L, Olaiyan I, Le Quesne G, Al Mazrou A (2003). Pediatric gastrointestinal basidiobolomycosis. Pediatr Infect Dis J.

[13] Fahimzad A, Karimi A, Tabatabaei SR, Zadeh MG (2006). Gastrointestinal basidiobolomycosis as a rare etiology of bowel obstruction. Turk J Med Sci.

[14] El Shabrawi MH, Kamal NM (2011). Gastrointestinal basidiobolomycosis in children: an overlooked emerging infection?. J Med Microbiol.

[15] Taghipour Zahir S, Sharahjin NS, Kargar S. Basidiobolomycosis a mysterious fungal infection mimic small intestinal and colonic tumour with renal insufficiency and ominous outcome. BMJ Case Rep. 2013 Jul 26;2013. doi:10.1136/bcr­2013­200244. 10.1136/bcr-2013-200244 PMC3736213 23893284

[16] Pasha TM, Leighton JA, Smilack JD, Heppell J, Colby TV, Kaufman L (1997). Basidiobolomycosis: an unusual fungal infection mimicking inflammatory bowel disease. Gastroenterol.

[17] Guarro J, Aguilar C, Pujol I (1999). In­vitro antifungal susceptibilities of Basidiobolus and Conidiobolus spp. strains. J Antimicrob Chemother.

[18] Yangco BG, Okafor JI, TeStrake D (1984). In vitro susceptibilities of human and wild­type isolates of Basidiobolus and Conidiobolus species. Antimicrob Agents Chemother.

